# 1-Cyclo­hexyl-2-(3-fur­yl)-1*H*-benzimidazole-5-carboxylic acid

**DOI:** 10.1107/S1600536809031213

**Published:** 2009-08-12

**Authors:** Sergey Dibrov, Sanjay Dutta, Thomas Hermann

**Affiliations:** a University of California, San Diego, 9500 Gilman Drive # 0358, La Jolla, CA 92093-0358, USA

## Abstract

The asymmetric unit of the title compound, C_18_H_18_N_2_O_3_, contains two mol­ecules. The fused rings of both mol­ecules are almost planar, with dihedral angles of 3.1 (1) and 2.8 (2)° between the fused rings. The furan rings are rotated by 43.85 (15) and −21.07 (9)° with respect to the planes of the attached bnzimidazole systems. In the crystal, mol­ecules are linked into infinite chains by inter­molecular O—H⋯N hydrogen bonds.

## Related literature

For general background, see Beaulieu *et al.* (2004*a* ▶). For the synthesis, see Beaulieu *et al.* (2004*b* ▶).
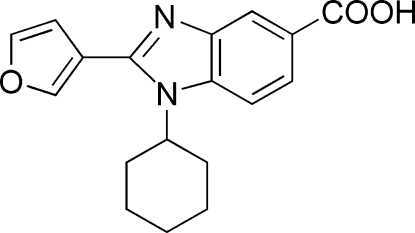


## Experimental

### 

#### Crystal data

C_18_H_18_N_2_O_3_
*M* *_r_* = 310.34
Monoclinic, 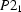

*a* = 9.1402 (3) Å
*b* = 11.2446 (3) Å
*c* = 15.6061 (5) Å
β = 101.334 (2)°
*V* = 1572.68 (8) Å^3^
*Z* = 4
Cu *K*α radiation
μ = 0.74 mm^−1^
*T* = 100 K
0.50 × 0.50 × 0.45 mm

#### Data collection

Bruker APEXII CCD area-detector diffractometer
Absorption correction: multi-scan (**SADABS**; Bruker, 2005 ▶) *T*_min_ = 0.710, *T*_max_ = 0.733
7859 measured reflections
4379 independent reflections
4006 reflections with *I* > 2σ(*I*)
*R*_int_ = 0.027

#### Refinement

*R*[*F* ^2^ > 2σ(*F* ^2^)] = 0.052
*wR*(*F* ^2^) = 0.143
*S* = 1.06
4379 reflections
423 parameters
1 restraint
H atoms treated by a mixture of independent and constrained refinement
Δρ_max_ = 0.73 e Å^−3^
Δρ_min_ = −0.29 e Å^−3^

### 

Data collection: *APEX2* (Bruker, 2005 ▶); cell refinement: *SAINT* (Bruker, 2005 ▶); data reduction: *SAINT*; program(s) used to solve structure: *SHELXS97* (Sheldrick, 2008 ▶); program(s) used to refine structure: *SHELXL97* (Sheldrick, 2008 ▶); molecular graphics: *SHELXTL* (Sheldrick, 2008 ▶); software used to prepare material for publication: *SHELXTL*.

## Supplementary Material

Crystal structure: contains datablocks I, global. DOI: 10.1107/S1600536809031213/rk2157sup1.cif

Structure factors: contains datablocks I. DOI: 10.1107/S1600536809031213/rk2157Isup2.hkl

Additional supplementary materials:  crystallographic information; 3D view; checkCIF report

## Figures and Tables

**Table 1:** Hydrogen-bond geometry (Å, °)

*D*—H⋯*A*	*D*—H	H⋯*A*	*D*⋯*A*	*D*—H⋯*A*
O2—H2⋯N2^i^	0.99 (5)	1.66 (5)	2.653 (3)	173 (4)
O4—H4⋯N4^ii^	0.75 (5)	1.94 (5)	2.653 (4)	159 (5)

Symmetry codes: (i) 
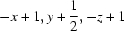
; (ii) 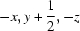.

**Fig. 1.**
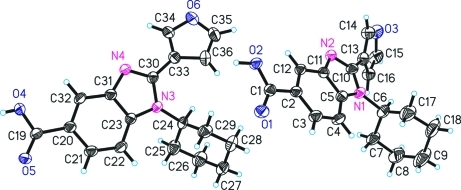
The asymmetric units of structure of title compound, with the atom–numbering scheme. The displacement ellipsoids are drawn at 50% probability level. H atoms are presented as a small spheres of arbitrary radius.

**Fig. 2.**
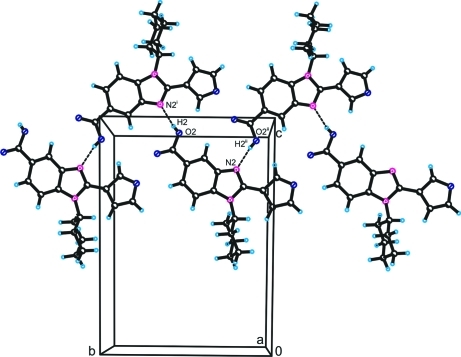
The crystal packing of one of the two molecules, viewed down the a axis, showing the molecules are linked along the b axis. Intermolecular hydrogen bonds are shown as dashed lines. Symmetry codes: (i) -x + 1, y + 1/2, -z + 1 and (ii) -x + 1, y - 1/2, -z + 1. Second molecule behaves similarly.

## supplementary crystallographic information

### Comment

The title compound is an allosteric inhibitor of the *RNA*–dependent *RNA* polymerase *NS*5*B* of hepatitis C virus (*HCV*) (Beaulieu *et al.*, 2004*a*). The inhibitor binds with low micromolar affinity (4.3 m*M*) to the *NS*5*B* polymerase and prevents replication of subgenomic *HCV* replicon in human cells (Beaulieu *et al.*, 2004*b*). The title compound was synthesized following the route described by Beaulieu *et al.*, (2004*b*). We report here the single–crystal *X*–ray structure.

Asymmetric unit of the title compound is composed of molecule 1 and molecule 2 (Fig. 1). They are linked together into infinite non–interacting chains by the intermolecular O2—H2···N2^i^ and O4—H4···N4^ii^ hydrogen bonds along the *b* axis (Fig. 2). Symmetry codes: (i) -*x* + 1, *y* + 1/2, -*z* + 1 and (ii) -*x*, *y* + 1/2, -*z*.

### Experimental

1-Cyclohexyl-2-(furan-3-yl)-1*H*-benzo[*d*]imidazole-5-carboxylic acid was prepared according to the literature procedure (Beaulieu *et al.*, 2004*b*). In a sample vial, 15 mg of compound was taken and dissolved in CH_2_Cl_2_–*DMSO*/ 9:1 mixture. Upon slow evaporation at 273 K within 2 months the crystals are formed as colourless blocks.

### Refinement

Acidic H2 and H4 hydrgen atoms were located in a Fourier difference map and refined freely with distances obtained for O2—H2 = 1.00 (5) and O4—H4 = 0.76 (5). Other H atoms were positioned geometrically and refined using a riding model with C—H = 0.95–0.99 Å and with *U*_iso_(H) = 1.2 *U*_eq_(C).

The highest density value is peak with a value of 0.73 e Å^-3^ at coordinates 0.5281 0.1307 0.9370 that is 1.21Å from atom H6 and 1.24Å from atom C17.

1455 Friedel pairs were merged.

### Crystal data

**Table:**

C_18_H_18_N_2_O_3_	*F*(000) = 656
*M**_r_* = 310.34	*D*_x_ = 1.311 Mg m^−^^3^
Monoclinic, *P*2_1_	Cu *K*α radiation, λ = 1.54178 Å
Hall symbol: P 2yb	Cell parameters from 5005 reflections
*a* = 9.1402 (3) Å	θ = 2.9–65.6°
*b* = 11.2446 (3) Å	µ = 0.74 mm^−^^1^
*c* = 15.6061 (5) Å	*T* = 100 K
β = 101.334 (2)°	Block, colourless
*V* = 1572.68 (8) Å^3^	0.50 × 0.50 × 0.45 mm
*Z* = 4	

### Data collection

**Table:**

Bruker APEXII CCD area-detector diffractometer	4379 independent reflections
Radiation source: fine-focus sealed tube	4006 reflections with *I* > 2σ(*I*)
graphite	*R*_int_ = 0.027
φ and ω scans	θ_max_ = 66.4°, θ_min_ = 2.9°
Absorption correction: multi-scan (*SADABS*; Bruker, 2005)	*h* = −10→10
*T*_min_ = 0.710, *T*_max_ = 0.733	*k* = −13→12
7859 measured reflections	*l* = −18→17

### Refinement

**Table:**

Refinement on *F*^2^	Primary atom site location: structure-invariant direct methods
Least-squares matrix: full	Secondary atom site location: difference Fourier map
*R*[*F*^2^ > 2σ(*F*^2^)] = 0.052	Hydrogen site location: inferred from neighbouring sites
*wR*(*F*^2^) = 0.143	H atoms treated by a mixture of independent and constrained refinement
*S* = 1.06	*w* = 1/[σ^2^(*F*_o_^2^) + (0.0919*P*)^2^ + 0.4805*P*] where *P* = (*F*_o_^2^ + 2*F*_c_^2^)/3
4379 reflections	(Δ/σ)_max_ = 0.007
423 parameters	Δρ_max_ = 0.73 e Å^−^^3^
1 restraint	Δρ_min_ = −0.29 e Å^−^^3^

### Special details

**Table:**

Geometry. All s.u.'s (except the s.u. in the dihedral angle between two l.s. planes) are estimated using the full covariance matrix. The cell s.u.'s are taken into account individually in the estimation of s.u.'s in distances, angles and torsion angles; correlations between s.u.'s in cell parameters are only used when they are defined by crystal symmetry. An approximate (isotropic) treatment of cell s.u.'s is used for estimating s.u.'s involving l.s. planes.
Refinement. Refinement of *F*^2^ against ALL reflections. The weighted *R*–factor *wR* and goodness of fit *S* are based on *F*^2^, conventional *R*–factors *R* are based on *F*, with *F* set to zero for negative *F*^2^. The threshold expression of *F*^2^ > σ(*F*^2^) is used only for calculating *R*–factors(gt) *etc*. and is not relevant to the choice of reflections for refinement. *R*–factors based on *F*^2^ are statistically about twice as large as those based on *F*, and *R*–factors based on ALL data will be even larger.

### Fractional atomic coordinates and isotropic or equivalent isotropic displacement parameters (Å^2^)

**Table:**

	*x*	*y*	*z*	*U*_iso_*/*U*_eq_	
O1	0.5638 (3)	0.67629 (19)	0.56838 (16)	0.0384 (6)	
O2	0.5668 (3)	0.5223 (2)	0.47898 (14)	0.0335 (5)	
H2	0.574 (5)	0.581 (4)	0.432 (3)	0.059 (13)*	
O3	0.3079 (3)	−0.1746 (2)	0.74288 (17)	0.0496 (7)	
N1	0.4370 (3)	0.2274 (3)	0.79176 (17)	0.0330 (6)	
N2	0.4291 (3)	0.1685 (2)	0.65399 (17)	0.0283 (6)	
C1	0.5558 (3)	0.5707 (3)	0.5547 (2)	0.0292 (7)	
C2	0.5294 (4)	0.4824 (3)	0.6213 (2)	0.0277 (7)	
C3	0.5382 (4)	0.5195 (3)	0.7078 (2)	0.0345 (8)	
H3	0.5628	0.6000	0.7224	0.041*	
C4	0.5126 (4)	0.4436 (3)	0.7724 (2)	0.0358 (8)	
H4B	0.5186	0.4696	0.8309	0.043*	
C5	0.4767 (4)	0.3249 (3)	0.7470 (2)	0.0297 (7)	
C6	0.4321 (5)	0.2198 (4)	0.8860 (2)	0.0465 (9)	
H6	0.4030	0.1356	0.8946	0.056*	
C7	0.3093 (4)	0.2922 (4)	0.9103 (2)	0.0448 (9)	
H7A	0.2132	0.2687	0.8728	0.054*	
H7B	0.3265	0.3773	0.8991	0.054*	
C8	0.2991 (6)	0.2766 (6)	1.0046 (3)	0.0717 (15)	
H8A	0.2319	0.3385	1.0203	0.086*	
H8B	0.2534	0.1983	1.0116	0.086*	
C9	0.4430 (6)	0.2838 (8)	1.0658 (3)	0.093 (2)	
H9A	0.4290	0.2613	1.1250	0.111*	
H9B	0.4793	0.3669	1.0684	0.111*	
C10	0.4080 (4)	0.1373 (3)	0.7332 (2)	0.0310 (7)	
C11	0.4698 (3)	0.2865 (3)	0.6616 (2)	0.0267 (7)	
C12	0.4959 (3)	0.3654 (3)	0.5968 (2)	0.0267 (7)	
H12	0.4909	0.3398	0.5383	0.032*	
C13	0.3512 (4)	0.0199 (3)	0.7503 (2)	0.0359 (8)	
C14	0.3900 (5)	−0.0844 (3)	0.7186 (3)	0.0432 (9)	
H14	0.4641	−0.0935	0.6842	0.052*	
C15	0.2130 (5)	−0.1246 (4)	0.7902 (3)	0.0476 (10)	
H15	0.1418	−0.1675	0.8149	0.057*	
C16	0.2339 (4)	−0.0080 (3)	0.7971 (2)	0.0396 (9)	
H16	0.1820	0.0464	0.8269	0.047*	
C17	0.5769 (5)	0.2333 (5)	0.9441 (3)	0.0608 (12)	
H17A	0.6121	0.3164	0.9421	0.073*	
H17B	0.6509	0.1803	0.9251	0.073*	
C18	0.5620 (5)	0.2013 (6)	1.0386 (3)	0.0738 (17)	
H18A	0.6591	0.2121	1.0790	0.089*	
H18B	0.5315	0.1172	1.0413	0.089*	
O4	−0.0250 (4)	1.0807 (2)	−0.01851 (16)	0.0440 (7)	
H4	−0.071 (5)	1.119 (4)	−0.053 (3)	0.047 (13)*	
O5	−0.0456 (3)	1.2233 (2)	0.07781 (15)	0.0367 (6)	
O6	0.1913 (3)	0.3434 (2)	0.23203 (17)	0.0481 (7)	
N3	0.1075 (3)	0.7453 (2)	0.30021 (16)	0.0274 (6)	
N4	0.1229 (3)	0.7088 (2)	0.16093 (17)	0.0294 (6)	
C19	−0.0173 (4)	1.1217 (3)	0.0617 (2)	0.0313 (7)	
C20	0.0290 (4)	1.0279 (3)	0.1289 (2)	0.0306 (7)	
C21	0.0299 (4)	1.0543 (3)	0.2180 (2)	0.0339 (8)	
H21	0.0115	1.1337	0.2340	0.041*	
C22	0.0563 (4)	0.9688 (3)	0.2813 (2)	0.0336 (8)	
H22	0.0563	0.9879	0.3406	0.040*	
C23	0.0834 (4)	0.8530 (3)	0.2568 (2)	0.0277 (7)	
C24	0.0788 (4)	0.7156 (3)	0.3880 (2)	0.0344 (8)	
H24	0.0636	0.6275	0.3874	0.041*	
C25	−0.0670 (4)	0.7689 (4)	0.4042 (2)	0.0447 (9)	
H25A	−0.0587	0.8566	0.4073	0.054*	
H25B	−0.1496	0.7481	0.3553	0.054*	
C26	−0.1004 (4)	0.7210 (4)	0.4894 (2)	0.0508 (10)	
H26A	−0.1911	0.7603	0.5016	0.061*	
H26B	−0.1207	0.6346	0.4834	0.061*	
C27	0.0277 (4)	0.7422 (4)	0.5646 (2)	0.0462 (9)	
H27A	0.0047	0.7054	0.6181	0.055*	
H27B	0.0401	0.8288	0.5750	0.055*	
C28	0.1737 (4)	0.6900 (4)	0.5466 (2)	0.0470 (10)	
H28A	0.2567	0.7105	0.5954	0.056*	
H28B	0.1657	0.6022	0.5432	0.056*	
C29	0.2072 (4)	0.7384 (4)	0.4609 (2)	0.0414 (9)	
H29A	0.2263	0.8250	0.4665	0.050*	
H29B	0.2978	0.6996	0.4483	0.050*	
C30	0.1327 (4)	0.6622 (3)	0.2394 (2)	0.0275 (7)	
C31	0.0913 (4)	0.8276 (3)	0.1692 (2)	0.0284 (7)	
C32	0.0629 (4)	0.9149 (3)	0.1045 (2)	0.0314 (7)	
H32	0.0669	0.8971	0.0455	0.038*	
C33	0.1669 (4)	0.5375 (3)	0.2578 (2)	0.0316 (8)	
C34	0.1464 (5)	0.4501 (3)	0.1967 (2)	0.0427 (9)	
H34	0.1056	0.4624	0.1365	0.051*	
C35	0.2435 (4)	0.3639 (3)	0.3181 (2)	0.0402 (9)	
H35	0.2833	0.3044	0.3594	0.048*	
C36	0.2317 (4)	0.4778 (3)	0.3373 (2)	0.0396 (9)	
H36	0.2607	0.5132	0.3933	0.047*	

### Atomic displacement parameters (Å^2^)

**Table:**

	*U*^11^	*U*^22^	*U*^33^	*U*^12^	*U*^13^	*U*^23^
O1	0.0570 (15)	0.0210 (11)	0.0376 (13)	−0.0062 (11)	0.0105 (11)	−0.0037 (10)
O2	0.0536 (14)	0.0232 (11)	0.0261 (12)	−0.0006 (10)	0.0141 (10)	0.0000 (10)
O3	0.0725 (19)	0.0356 (14)	0.0444 (15)	−0.0083 (13)	0.0205 (13)	0.0069 (12)
N1	0.0425 (15)	0.0356 (15)	0.0242 (13)	0.0028 (13)	0.0148 (11)	0.0024 (12)
N2	0.0379 (14)	0.0243 (13)	0.0250 (14)	−0.0011 (11)	0.0117 (11)	0.0048 (11)
C1	0.0297 (16)	0.0297 (17)	0.0278 (17)	−0.0007 (13)	0.0047 (13)	−0.0006 (13)
C2	0.0328 (16)	0.0237 (15)	0.0275 (16)	0.0003 (13)	0.0079 (13)	−0.0013 (13)
C3	0.0452 (19)	0.0275 (16)	0.0318 (17)	−0.0029 (15)	0.0103 (14)	−0.0042 (15)
C4	0.048 (2)	0.0358 (19)	0.0259 (17)	−0.0027 (16)	0.0119 (14)	−0.0061 (15)
C5	0.0321 (17)	0.0330 (17)	0.0270 (16)	0.0006 (14)	0.0132 (13)	0.0022 (14)
C6	0.059 (2)	0.059 (2)	0.0251 (18)	0.012 (2)	0.0184 (16)	0.0053 (18)
C7	0.044 (2)	0.065 (3)	0.0292 (18)	0.0042 (19)	0.0172 (15)	−0.0026 (18)
C8	0.080 (3)	0.104 (4)	0.039 (2)	0.023 (3)	0.031 (2)	0.001 (3)
C9	0.065 (3)	0.181 (7)	0.036 (2)	0.018 (4)	0.020 (2)	−0.018 (3)
C10	0.0334 (17)	0.0314 (17)	0.0308 (18)	0.0010 (14)	0.0129 (14)	−0.0003 (14)
C11	0.0290 (15)	0.0250 (15)	0.0272 (16)	0.0008 (13)	0.0078 (12)	0.0015 (13)
C12	0.0337 (17)	0.0238 (15)	0.0239 (15)	−0.0008 (13)	0.0090 (13)	0.0002 (13)
C13	0.0413 (19)	0.0357 (18)	0.0326 (18)	−0.0024 (16)	0.0116 (14)	0.0075 (15)
C14	0.059 (2)	0.0337 (19)	0.042 (2)	−0.0020 (18)	0.0239 (17)	0.0069 (16)
C15	0.059 (2)	0.049 (2)	0.040 (2)	−0.0145 (19)	0.0240 (18)	0.0060 (18)
C16	0.044 (2)	0.039 (2)	0.0387 (19)	−0.0033 (16)	0.0165 (16)	0.0024 (16)
C17	0.053 (2)	0.087 (3)	0.046 (2)	0.002 (2)	0.0174 (19)	−0.006 (2)
C18	0.052 (2)	0.140 (5)	0.029 (2)	0.014 (3)	0.0087 (18)	−0.004 (3)
O4	0.080 (2)	0.0250 (12)	0.0266 (14)	0.0108 (13)	0.0106 (13)	0.0031 (11)
O5	0.0565 (15)	0.0239 (12)	0.0341 (13)	0.0049 (11)	0.0194 (10)	−0.0001 (10)
O6	0.0618 (17)	0.0331 (13)	0.0502 (16)	0.0133 (12)	0.0132 (13)	0.0068 (12)
N3	0.0308 (13)	0.0306 (13)	0.0229 (13)	0.0004 (11)	0.0102 (11)	0.0017 (11)
N4	0.0412 (15)	0.0223 (13)	0.0264 (14)	−0.0007 (11)	0.0110 (11)	0.0007 (11)
C19	0.0425 (19)	0.0275 (18)	0.0264 (17)	−0.0039 (15)	0.0128 (14)	−0.0035 (14)
C20	0.0419 (18)	0.0232 (15)	0.0286 (16)	−0.0032 (14)	0.0119 (13)	−0.0026 (13)
C21	0.049 (2)	0.0245 (17)	0.0315 (18)	−0.0046 (14)	0.0153 (15)	−0.0053 (14)
C22	0.046 (2)	0.0330 (17)	0.0237 (16)	−0.0020 (15)	0.0111 (14)	−0.0031 (15)
C23	0.0319 (17)	0.0284 (16)	0.0252 (16)	−0.0003 (13)	0.0115 (13)	0.0021 (13)
C24	0.0462 (19)	0.0370 (18)	0.0234 (16)	0.0058 (16)	0.0149 (14)	0.0087 (15)
C25	0.0390 (19)	0.060 (2)	0.037 (2)	0.0021 (18)	0.0117 (15)	0.0075 (18)
C26	0.045 (2)	0.068 (3)	0.044 (2)	−0.002 (2)	0.0188 (17)	0.003 (2)
C27	0.055 (2)	0.056 (2)	0.0303 (18)	0.0059 (19)	0.0165 (16)	0.0051 (18)
C28	0.050 (2)	0.060 (3)	0.0288 (19)	−0.003 (2)	0.0045 (16)	0.0063 (18)
C29	0.0390 (19)	0.050 (2)	0.0376 (19)	0.0004 (17)	0.0135 (15)	−0.0036 (17)
C30	0.0303 (16)	0.0280 (16)	0.0266 (16)	−0.0033 (13)	0.0115 (13)	0.0032 (13)
C31	0.0356 (17)	0.0237 (15)	0.0279 (16)	−0.0019 (13)	0.0111 (13)	−0.0016 (13)
C32	0.0461 (19)	0.0281 (16)	0.0220 (16)	−0.0053 (15)	0.0115 (13)	−0.0027 (13)
C33	0.0312 (17)	0.0310 (17)	0.0356 (18)	0.0018 (14)	0.0137 (14)	0.0069 (15)
C34	0.063 (2)	0.0302 (18)	0.038 (2)	0.0144 (17)	0.0168 (17)	0.0068 (16)
C35	0.0377 (19)	0.037 (2)	0.046 (2)	0.0070 (15)	0.0099 (16)	0.0137 (17)
C36	0.041 (2)	0.040 (2)	0.0349 (19)	0.0012 (16)	0.0021 (15)	0.0108 (17)

### Geometric parameters (Å, °)

**Table:**

O1—C1	1.207 (4)	O4—C19	1.324 (4)
O2—C1	1.322 (4)	O4—H4	0.75 (5)
O2—H2	0.99 (5)	O5—C19	1.208 (4)
O3—C14	1.359 (5)	O6—C34	1.350 (4)
O3—C15	1.366 (5)	O6—C35	1.354 (5)
N1—C10	1.355 (4)	N3—C23	1.383 (4)
N1—C5	1.387 (4)	N3—C30	1.383 (4)
N1—C6	1.482 (4)	N3—C24	1.482 (4)
N2—C10	1.335 (4)	N4—C30	1.319 (4)
N2—C11	1.377 (4)	N4—C31	1.378 (4)
C1—C2	1.492 (5)	C19—C20	1.489 (5)
C2—C12	1.387 (4)	C20—C32	1.378 (5)
C2—C3	1.400 (4)	C20—C21	1.421 (5)
C3—C4	1.376 (5)	C21—C22	1.366 (5)
C3—H3	0.9500	C21—H21	0.9500
C4—C5	1.411 (5)	C22—C23	1.393 (5)
C4—H4B	0.9500	C22—H22	0.9500
C5—C11	1.391 (4)	C23—C31	1.413 (4)
C6—C17	1.459 (6)	C24—C29	1.488 (5)
C6—C7	1.495 (6)	C24—C25	1.528 (5)
C6—H6	1.0000	C24—H24	1.0000
C7—C8	1.504 (5)	C25—C26	1.518 (5)
C7—H7A	0.9900	C25—H25A	0.9900
C7—H7B	0.9900	C25—H25B	0.9900
C8—C9	1.468 (7)	C26—C27	1.505 (5)
C8—H8A	0.9900	C26—H26A	0.9900
C8—H8B	0.9900	C26—H26B	0.9900
C9—C18	1.550 (8)	C27—C28	1.533 (6)
C9—H9A	0.9900	C27—H27A	0.9900
C9—H9B	0.9900	C27—H27B	0.9900
C10—C13	1.462 (5)	C28—C29	1.530 (5)
C11—C12	1.401 (4)	C28—H28A	0.9900
C12—H12	0.9500	C28—H28B	0.9900
C13—C14	1.346 (5)	C29—H29A	0.9900
C13—C16	1.447 (5)	C29—H29B	0.9900
C14—H14	0.9500	C30—C33	1.452 (5)
C15—C16	1.326 (6)	C31—C32	1.396 (5)
C15—H15	0.9500	C32—H32	0.9500
C16—H16	0.9500	C33—C34	1.358 (5)
C17—C18	1.549 (6)	C33—C36	1.433 (4)
C17—H17A	0.9900	C34—H34	0.9500
C17—H17B	0.9900	C35—C36	1.324 (5)
C18—H18A	0.9900	C35—H35	0.9500
C18—H18B	0.9900	C36—H36	0.9500
			
C1—O2—H2	114 (3)	C19—O4—H4	114 (3)
C14—O3—C15	106.7 (3)	C34—O6—C35	105.9 (3)
C10—N1—C5	106.7 (3)	C23—N3—C30	106.7 (2)
C10—N1—C6	125.9 (3)	C23—N3—C24	127.5 (3)
C5—N1—C6	127.4 (3)	C30—N3—C24	124.4 (3)
C10—N2—C11	105.0 (3)	C30—N4—C31	106.0 (3)
O1—C1—O2	123.4 (3)	O5—C19—O4	123.5 (3)
O1—C1—C2	122.9 (3)	O5—C19—C20	124.5 (3)
O2—C1—C2	113.6 (3)	O4—C19—C20	111.9 (3)
C12—C2—C3	121.1 (3)	C32—C20—C21	120.4 (3)
C12—C2—C1	119.7 (3)	C32—C20—C19	120.5 (3)
C3—C2—C1	119.2 (3)	C21—C20—C19	119.1 (3)
C4—C3—C2	122.3 (3)	C22—C21—C20	121.8 (3)
C4—C3—H3	118.8	C22—C21—H21	119.1
C2—C3—H3	118.8	C20—C21—H21	119.1
C3—C4—C5	116.4 (3)	C21—C22—C23	118.4 (3)
C3—C4—H4B	121.8	C21—C22—H22	120.8
C5—C4—H4B	121.8	C23—C22—H22	120.8
N1—C5—C11	105.6 (3)	N3—C23—C22	134.8 (3)
N1—C5—C4	132.5 (3)	N3—C23—C31	105.3 (3)
C11—C5—C4	121.8 (3)	C22—C23—C31	119.9 (3)
C17—C6—N1	114.3 (3)	N3—C24—C29	114.3 (3)
C17—C6—C7	114.9 (4)	N3—C24—C25	112.6 (3)
N1—C6—C7	113.0 (3)	C29—C24—C25	112.8 (3)
C17—C6—H6	104.4	N3—C24—H24	105.4
N1—C6—H6	104.4	C29—C24—H24	105.4
C7—C6—H6	104.4	C25—C24—H24	105.4
C6—C7—C8	112.3 (3)	C26—C25—C24	109.8 (3)
C6—C7—H7A	109.2	C26—C25—H25A	109.7
C8—C7—H7A	109.2	C24—C25—H25A	109.7
C6—C7—H7B	109.2	C26—C25—H25B	109.7
C8—C7—H7B	109.2	C24—C25—H25B	109.7
H7A—C7—H7B	107.9	H25A—C25—H25B	108.2
C9—C8—C7	114.2 (4)	C27—C26—C25	111.5 (3)
C9—C8—H8A	108.7	C27—C26—H26A	109.3
C7—C8—H8A	108.7	C25—C26—H26A	109.3
C9—C8—H8B	108.7	C27—C26—H26B	109.3
C7—C8—H8B	108.7	C25—C26—H26B	109.3
H8A—C8—H8B	107.6	H26A—C26—H26B	108.0
C8—C9—C18	112.1 (5)	C26—C27—C28	111.6 (3)
C8—C9—H9A	109.2	C26—C27—H27A	109.3
C18—C9—H9A	109.2	C28—C27—H27A	109.3
C8—C9—H9B	109.2	C26—C27—H27B	109.3
C18—C9—H9B	109.2	C28—C27—H27B	109.3
H9A—C9—H9B	107.9	H27A—C27—H27B	108.0
N2—C10—N1	112.6 (3)	C29—C28—C27	110.8 (3)
N2—C10—C13	121.8 (3)	C29—C28—H28A	109.5
N1—C10—C13	125.5 (3)	C27—C28—H28A	109.5
N2—C11—C5	110.1 (3)	C29—C28—H28B	109.5
N2—C11—C12	129.0 (3)	C27—C28—H28B	109.5
C5—C11—C12	120.8 (3)	H28A—C28—H28B	108.1
C2—C12—C11	117.5 (3)	C24—C29—C28	110.1 (3)
C2—C12—H12	121.2	C24—C29—H29A	109.6
C11—C12—H12	121.2	C28—C29—H29A	109.6
C14—C13—C16	105.7 (3)	C24—C29—H29B	109.6
C14—C13—C10	126.2 (3)	C28—C29—H29B	109.6
C16—C13—C10	127.8 (3)	H29A—C29—H29B	108.1
C13—C14—O3	110.4 (3)	N4—C30—N3	112.2 (3)
C13—C14—H14	124.8	N4—C30—C33	122.7 (3)
O3—C14—H14	124.8	N3—C30—C33	125.1 (3)
C16—C15—O3	110.7 (4)	N4—C31—C32	128.8 (3)
C16—C15—H15	124.6	N4—C31—C23	109.7 (3)
O3—C15—H15	124.6	C32—C31—C23	121.4 (3)
C15—C16—C13	106.4 (4)	C20—C32—C31	117.9 (3)
C15—C16—H16	126.8	C20—C32—H32	121.1
C13—C16—H16	126.8	C31—C32—H32	121.1
C6—C17—C18	109.2 (4)	C34—C33—C36	104.2 (3)
C6—C17—H17A	109.8	C34—C33—C30	124.4 (3)
C18—C17—H17A	109.8	C36—C33—C30	131.3 (3)
C6—C17—H17B	109.8	O6—C34—C33	111.5 (3)
C18—C17—H17B	109.8	O6—C34—H34	124.2
H17A—C17—H17B	108.3	C33—C34—H34	124.2
C17—C18—C9	108.6 (4)	C36—C35—O6	111.1 (3)
C17—C18—H18A	110.0	C36—C35—H35	124.4
C9—C18—H18A	110.0	O6—C35—H35	124.4
C17—C18—H18B	110.0	C35—C36—C33	107.2 (3)
C9—C18—H18B	110.0	C35—C36—H36	126.4
H18A—C18—H18B	108.3	C33—C36—H36	126.4

### Hydrogen-bond geometry (Å, °)

**Table:**

*D*—H···*A*	*D*—H	H···*A*	*D*···*A*	*D*—H···*A*
O2—H2···N2^i^	0.99 (5)	1.66 (5)	2.653 (3)	173 (4)
O4—H4···N4^ii^	0.75 (5)	1.94 (5)	2.653 (4)	159 (5)

Symmetry codes: (i) −*x*+1, *y*+1/2, −*z*+1; (ii) −*x*, *y*+1/2, −*z*.
